# 2-Methyl-*N*-(4-nitro­benzo­yl)benzene­sulfonamide

**DOI:** 10.1107/S1600536811009846

**Published:** 2011-03-19

**Authors:** P. A. Suchetan, Sabine Foro, B. Thimme Gowda

**Affiliations:** a Department of Chemistry, Mangalore University, Mangalagangotri 574 199, Mangalore, India; b Institute of Materials Science, Darmstadt University of Technology, Petersenstrasse 23, D-64287 Darmstadt, Germany

## Abstract

In the title compound, C_14_H_12_N_2_O_5_S, the conformation of the N—C bond in the C—SO_2_—NH—C(O) segment has *gauche* torsions with respect to the S=O bonds. The mol­ecule is twisted at the S atom, the C—S(O_2_)—NH—C(O) torsion angle being 61.8 (5)°. The dihedral angle between the sulfonyl benzene ring and the —SO_2_—NH—C—O segment is 86.8 (2)° and that between the sulfonyl and the benzoyl benzene rings is 83.8 (2)°. In the crystal, mol­ecules are linked into zigzag chains along the *a* axis *via* N—H⋯O hydrogen bonds.

## Related literature

For our study of the effect of substituents on the structures of *N*-(ar­yl)-amides, see: Gowda *et al.* (2000 ▶), on the structures of *N*-(ar­yl)-methane­sulfonamides, see: Gowda *et al.* (2007 ▶) and on the structures of *N*-(*p*-substituted-benzo­yl)-*p*-substituted-benzene­sulfon­amides, see: Suchetan *et al.* (2010 ▶, 2011 ▶).
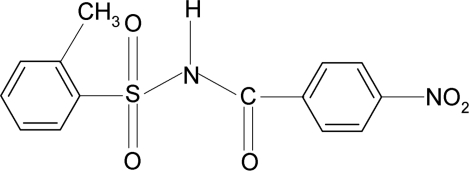


## Experimental

### 

#### Crystal data

C_14_H_12_N_2_O_5_S
*M* *_r_* = 320.32
Monoclinic, 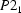

*a* = 11.088 (2) Å
*b* = 5.3490 (7) Å
*c* = 12.344 (2) Å
β = 104.45 (2)°
*V* = 709.0 (2) Å^3^
*Z* = 2
Mo *K*α radiation
μ = 0.26 mm^−1^
*T* = 293 K
0.36 × 0.14 × 0.08 mm

#### Data collection

Oxford Diffraction Xcalibur diffractometer with a Sapphire CCD detector
Absorption correction: multi-scan (*CrysAlis RED*; Oxford Diffraction, 2009 ▶) *T*_min_ = 0.914, *T*_max_ = 0.980
2485 measured reflections
1912 independent reflections
1701 reflections with *I* > 2σ(*I*)
*R*_int_ = 0.031

#### Refinement

*R*[*F* ^2^ > 2σ(*F* ^2^)] = 0.058
*wR*(*F* ^2^) = 0.146
*S* = 1.22
1912 reflections
203 parameters
2 restraints
H atoms treated by a mixture of independent and constrained refinement
Δρ_max_ = 0.40 e Å^−3^
Δρ_min_ = −0.49 e Å^−3^
Absolute structure: Flack (1983 ▶), 295 Friedel pairs
Flack parameter: 0.2 (2)

### 

Data collection: *CrysAlis CCD* (Oxford Diffraction, 2009 ▶); cell refinement: *CrysAlis RED* (Oxford Diffraction, 2009 ▶); data reduction: *CrysAlis RED*; program(s) used to solve structure: *SHELXS97* (Sheldrick, 2008 ▶); program(s) used to refine structure: *SHELXL97* (Sheldrick, 2008 ▶); molecular graphics: *PLATON* (Spek, 2009 ▶); software used to prepare material for publication: *SHELXL97*.

## Supplementary Material

Crystal structure: contains datablocks I, global. DOI: 10.1107/S1600536811009846/bq2287sup1.cif

Structure factors: contains datablocks I. DOI: 10.1107/S1600536811009846/bq2287Isup2.hkl

Additional supplementary materials:  crystallographic information; 3D view; checkCIF report

## Figures and Tables

**Table 1:** Hydrogen-bond geometry (Å, °)

*D*—H⋯*A*	*D*—H	H⋯*A*	*D*⋯*A*	*D*—H⋯*A*
N1—H1*N*⋯O2^i^	0.87 (3)	2.12 (4)	2.992 (6)	174 (5)

Symmetry code: (i) 
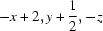
.

**Fig. 1.**
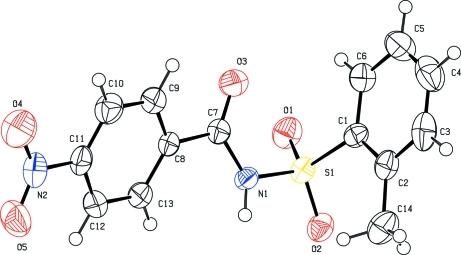
Molecular structure of the title compound, showing the atom labeling scheme. Displacement ellipsoids are drawn at the 50% probability level.

**Fig. 2.**
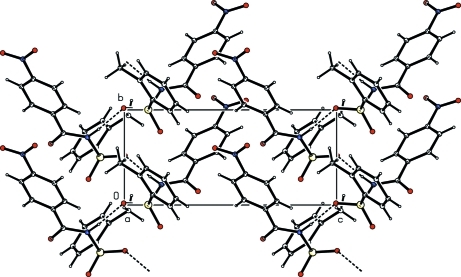
Molecular packing in the title compound. Hydrogen bonds are shown as dashed lines.

## supplementary crystallographic information

### Comment

The amide and sulfonamide moieties are important constituents of many biologically significant compounds. As a part of studying the effect of substituents on the structures of this class of compounds (Gowda *et al.*, 2000, 2007; Suchetan *et al.*, 2010, 2011), the structure of 2-methyl-*N*-(4-nitrobenzoyl)-benzenesulfonamide (I) has been determined (Fig.1). The conformation of the N—C bond in the C—SO_2_—NH—C(O) segment has *gauche* torsions with respect to the S═O bonds. Further, the N—H bond in the C—SO_2_—NH—C(O) segment is *anti* to the C=O bond, similar to those observed in 2-methyl-*N*-(4-chlorobenzoyl)-benzenesulfonamide (II) (Suchetan *et al.*, 2010) and 4-Methyl-*N*-(4-nitrobenzoyl)- benzenesulfonamide (III) (Suchetan *et al.*, 2011).

The molecules are twisted at the *S* atoms with the C—S(O_2_)—NH—C(O) torsional angle of 61.8 (5)°, compared to the values of -54.2 (2)° and 63.8 (2)°, in the two independent molecules of (II) and 58.7 (3)° in (III).

The dihedral angle between the sulfonyl benzene ring and the —SO_2_—NH—C—O segment is 86.8 (2)°, compared to the values of 85.0 (1)° (molecule 1) & 87.0 (1)° (molecule 2) in (II) and 81.5 (2)° in (III).

The dihedral angle between the sulfonyl and the benzoyl benzene rings is 83.8 (2)°, compared to the values of 89.4 (1)° (molecule 1) and 82.1 (1)° (molecule 2) in (II) and 89.8 (1)° in (III).

The packing of molecules in the crystal linked by of N—H···O hydrogen bonds (Table 1) is shown in Fig. 2.

### Experimental

The title compound was prepared by refluxing a mixture of 4-nitrobenzoic acid, 2-methylbenzenesulfonamide and phosphorous oxychloride for 3 h on a water bath. The resultant mixture was cooled and poured into ice cold water. The solid obtained was filtered, washed thoroughly with water and then dissolved in sodium bicarbonate solution. The compound was later reprecipitated by acidifying the filtered solution with dilute HCl. It was filtered, dried and recrystallized.

Rod like colorless single crystals of the title compound used in x-ray diffraction studies were obtained by slow evaporation of its toluene solution at room temperature.

### Refinement

The H atom of the NH group was located in a difference map and later restrained to N—H = 0.86 (4) Å. The other H atoms were positioned with idealized geometry using a riding model with the aromatic C—H distance = 0.93 Å and methyl C—H = 0.96 Å. All H atoms were refined with isotropic displacement parameters (set to 1.2 times of the *U*_eq_ of the parent atom).

### Crystal data

**Table:**

C_14_H_12_N_2_O_5_S	*F*(000) = 332
*M**_r_* = 320.32	*D*_x_ = 1.501 Mg m^−^^3^
Monoclinic, *P*2_1_	Mo *K*α radiation, λ = 0.71073 Å
Hall symbol: P 2yb	Cell parameters from 1591 reflections
*a* = 11.088 (2) Å	θ = 2.9–28.0°
*b* = 5.3490 (7) Å	µ = 0.26 mm^−^^1^
*c* = 12.344 (2) Å	*T* = 293 K
β = 104.45 (2)°	Rod, colorless
*V* = 709.0 (2) Å^3^	0.36 × 0.14 × 0.08 mm
*Z* = 2	

### Data collection

**Table:**

Oxford Diffraction Xcalibur diffractometer with a Sapphire CCD detector	1912 independent reflections
Radiation source: fine-focus sealed tube	1701 reflections with *I* > 2σ(*I*)
graphite	*R*_int_ = 0.031
Rotation method data acquisition using ω and φ scans	θ_max_ = 26.4°, θ_min_ = 2.9°
Absorption correction: multi-scan (*CrysAlis RED*; Oxford Diffraction, 2009)	*h* = −11→13
*T*_min_ = 0.914, *T*_max_ = 0.980	*k* = −6→2
2485 measured reflections	*l* = −15→5

### Refinement

**Table:**

Refinement on *F*^2^	Secondary atom site location: difference Fourier map
Least-squares matrix: full	Hydrogen site location: inferred from neighbouring sites
*R*[*F*^2^ > 2σ(*F*^2^)] = 0.058	H atoms treated by a mixture of independent and constrained refinement
*wR*(*F*^2^) = 0.146	*w* = 1/[σ^2^(*F*_o_^2^) + (0.0445*P*)^2^ + 1.1687*P*] where *P* = (*F*_o_^2^ + 2*F*_c_^2^)/3
*S* = 1.22	(Δ/σ)_max_ < 0.001
1912 reflections	Δρ_max_ = 0.40 e Å^−^^3^
203 parameters	Δρ_min_ = −0.49 e Å^−^^3^
2 restraints	Absolute structure: Flack (1983), 295 Friedel pairs
Primary atom site location: structure-invariant direct methods	Flack parameter: 0.2 (2)

### Special details

**Table:**

Experimental. CrysAlis RED (Oxford Diffraction, 2009) Empirical absorption correction using spherical harmonics, implemented in SCALE3 ABSPACK scaling algorithm.
Geometry. All e.s.d.'s (except the e.s.d. in the dihedral angle between two l.s. planes) are estimated using the full covariance matrix. The cell e.s.d.'s are taken into account individually in the estimation of e.s.d.'s in distances, angles and torsion angles; correlations between e.s.d.'s in cell parameters are only used when they are defined by crystal symmetry. An approximate (isotropic) treatment of cell e.s.d.'s is used for estimating e.s.d.'s involving l.s. planes.
Refinement. Refinement of *F*^2^ against ALL reflections. The weighted *R*-factor *wR* and goodness of fit *S* are based on *F*^2^, conventional *R*-factors *R* are based on *F*, with *F* set to zero for negative *F*^2^. The threshold expression of *F*^2^ > σ(*F*^2^) is used only for calculating *R*-factors(gt) *etc*. and is not relevant to the choice of reflections for refinement. *R*-factors based on *F*^2^ are statistically about twice as large as those based on *F*, and *R*- factors based on ALL data will be even larger.

### Fractional atomic coordinates and isotropic or equivalent isotropic displacement parameters (Å^2^)

**Table:**

	*x*	*y*	*z*	*U*_iso_*/*U*_eq_	
C1	0.7626 (5)	0.1060 (12)	0.1331 (4)	0.0342 (12)	
C2	0.6994 (5)	0.3107 (13)	0.0729 (5)	0.0423 (14)	
C3	0.5918 (6)	0.3851 (16)	0.1032 (6)	0.0584 (19)	
H3	0.5454	0.5171	0.0649	0.070*	
C4	0.5522 (6)	0.2708 (18)	0.1870 (6)	0.064 (2)	
H4	0.4819	0.3312	0.2066	0.076*	
C5	0.6146 (6)	0.0673 (17)	0.2430 (6)	0.062 (2)	
H5	0.5852	−0.0150	0.2977	0.075*	
C6	0.7216 (5)	−0.0105 (17)	0.2157 (4)	0.0472 (14)	
H6	0.7666	−0.1440	0.2540	0.057*	
C7	1.0346 (5)	0.2574 (12)	0.2879 (4)	0.0366 (13)	
C8	1.1292 (4)	0.4580 (11)	0.3348 (4)	0.0328 (14)	
C9	1.1242 (5)	0.5551 (15)	0.4365 (4)	0.0476 (18)	
H9	1.0667	0.4932	0.4732	0.057*	
C10	1.2052 (6)	0.7466 (14)	0.4848 (5)	0.0488 (16)	
H10	1.2013	0.8180	0.5526	0.059*	
C11	1.2906 (5)	0.8261 (13)	0.4292 (5)	0.0409 (14)	
C12	1.2986 (5)	0.7304 (14)	0.3293 (5)	0.0464 (16)	
H12	1.3574	0.7902	0.2936	0.056*	
C13	1.2161 (5)	0.5394 (15)	0.2817 (4)	0.0451 (17)	
H13	1.2205	0.4681	0.2140	0.054*	
C14	0.7364 (6)	0.4421 (13)	−0.0192 (5)	0.0554 (18)	
H14A	0.7156	0.3408	−0.0855	0.067*	
H14B	0.8245	0.4721	0.0015	0.067*	
H14C	0.6929	0.5986	−0.0335	0.067*	
N1	1.0112 (4)	0.2138 (9)	0.1743 (3)	0.0319 (10)	
H1N	1.031 (5)	0.298 (11)	0.121 (4)	0.038*	
N2	1.3751 (4)	1.0337 (13)	0.4783 (4)	0.0491 (13)	
O1	0.9359 (4)	−0.2274 (9)	0.1691 (3)	0.0492 (11)	
O2	0.9059 (3)	0.0189 (10)	−0.0029 (3)	0.0428 (9)	
O3	0.9818 (4)	0.1447 (10)	0.3468 (3)	0.0539 (13)	
O4	1.3758 (5)	1.0989 (12)	0.5732 (4)	0.0753 (16)	
O5	1.4394 (4)	1.1259 (11)	0.4219 (4)	0.0690 (15)	
S1	0.90691 (11)	0.0026 (3)	0.11283 (10)	0.0351 (3)	

### Atomic displacement parameters (Å^2^)

**Table:**

	*U*^11^	*U*^22^	*U*^33^	*U*^12^	*U*^13^	*U*^23^
C1	0.037 (3)	0.039 (3)	0.026 (2)	−0.002 (3)	0.0066 (19)	−0.006 (2)
C2	0.039 (3)	0.043 (4)	0.040 (3)	−0.004 (3)	0.000 (2)	−0.005 (3)
C3	0.037 (3)	0.062 (5)	0.068 (4)	0.008 (3)	−0.003 (3)	−0.009 (4)
C4	0.044 (3)	0.082 (6)	0.068 (5)	−0.008 (4)	0.021 (3)	−0.025 (5)
C5	0.051 (3)	0.081 (7)	0.059 (4)	−0.015 (4)	0.021 (3)	−0.003 (4)
C6	0.042 (3)	0.054 (4)	0.044 (3)	−0.006 (4)	0.007 (2)	−0.001 (4)
C7	0.031 (3)	0.041 (4)	0.036 (3)	0.001 (3)	0.005 (2)	0.005 (3)
C8	0.027 (2)	0.039 (4)	0.031 (2)	0.001 (2)	0.0022 (18)	0.005 (2)
C9	0.038 (3)	0.068 (6)	0.036 (3)	−0.006 (3)	0.008 (2)	−0.002 (3)
C10	0.050 (3)	0.053 (4)	0.041 (3)	0.001 (3)	0.007 (3)	−0.011 (3)
C11	0.031 (3)	0.044 (4)	0.041 (3)	0.004 (3)	−0.002 (2)	0.004 (3)
C12	0.035 (3)	0.061 (5)	0.043 (3)	−0.010 (3)	0.008 (2)	0.000 (3)
C13	0.038 (3)	0.065 (5)	0.031 (2)	−0.008 (3)	0.006 (2)	−0.005 (3)
C14	0.064 (4)	0.044 (5)	0.052 (3)	0.007 (3)	0.001 (3)	0.005 (3)
N1	0.036 (2)	0.032 (3)	0.026 (2)	−0.004 (2)	0.0047 (17)	0.0006 (19)
N2	0.036 (2)	0.046 (4)	0.057 (3)	0.003 (3)	−0.002 (2)	−0.006 (3)
O1	0.052 (2)	0.039 (3)	0.052 (2)	0.007 (2)	0.0060 (19)	0.006 (2)
O2	0.0432 (19)	0.047 (3)	0.0359 (17)	0.002 (2)	0.0052 (14)	−0.006 (2)
O3	0.054 (2)	0.066 (3)	0.040 (2)	−0.015 (2)	0.0095 (18)	0.003 (2)
O4	0.083 (4)	0.079 (4)	0.059 (3)	−0.020 (3)	0.010 (2)	−0.027 (3)
O5	0.053 (3)	0.070 (4)	0.083 (3)	−0.020 (3)	0.015 (2)	−0.002 (3)
S1	0.0365 (6)	0.0337 (7)	0.0330 (6)	0.0020 (7)	0.0043 (4)	−0.0006 (7)

### Geometric parameters (Å, °)

**Table:**

C1—C6	1.366 (8)	C9—H9	0.9300
C1—C2	1.408 (9)	C10—C11	1.369 (9)
C1—S1	1.769 (5)	C10—H10	0.9300
C2—C3	1.395 (9)	C11—C12	1.359 (8)
C2—C14	1.479 (8)	C11—N2	1.481 (9)
C3—C4	1.365 (10)	C12—C13	1.399 (9)
C3—H3	0.9300	C12—H12	0.9300
C4—C5	1.380 (12)	C13—H13	0.9300
C4—H4	0.9300	C14—H14A	0.9600
C5—C6	1.377 (8)	C14—H14B	0.9600
C5—H5	0.9300	C14—H14C	0.9600
C6—H6	0.9300	N1—S1	1.659 (5)
C7—O3	1.202 (6)	N1—H1N	0.87 (3)
C7—N1	1.381 (6)	N2—O5	1.219 (7)
C7—C8	1.511 (8)	N2—O4	1.220 (6)
C8—C13	1.366 (7)	O1—S1	1.410 (5)
C8—C9	1.371 (7)	O2—S1	1.428 (3)
C9—C10	1.395 (9)		
			
C6—C1—C2	122.3 (5)	C9—C10—H10	121.1
C6—C1—S1	116.4 (5)	C12—C11—C10	123.3 (6)
C2—C1—S1	121.1 (4)	C12—C11—N2	118.4 (5)
C3—C2—C1	115.2 (6)	C10—C11—N2	118.3 (5)
C3—C2—C14	119.4 (6)	C11—C12—C13	118.2 (6)
C1—C2—C14	125.4 (5)	C11—C12—H12	120.9
C4—C3—C2	122.3 (7)	C13—C12—H12	120.9
C4—C3—H3	118.9	C8—C13—C12	119.8 (5)
C2—C3—H3	118.9	C8—C13—H13	120.1
C3—C4—C5	121.2 (7)	C12—C13—H13	120.1
C3—C4—H4	119.4	C2—C14—H14A	109.5
C5—C4—H4	119.4	C2—C14—H14B	109.5
C6—C5—C4	118.0 (7)	H14A—C14—H14B	109.5
C6—C5—H5	121.0	C2—C14—H14C	109.5
C4—C5—H5	121.0	H14A—C14—H14C	109.5
C1—C6—C5	120.9 (7)	H14B—C14—H14C	109.5
C1—C6—H6	119.6	C7—N1—S1	120.8 (4)
C5—C6—H6	119.6	C7—N1—H1N	132 (4)
O3—C7—N1	122.2 (5)	S1—N1—H1N	106 (4)
O3—C7—C8	121.4 (5)	O5—N2—O4	124.6 (6)
N1—C7—C8	116.5 (4)	O5—N2—C11	118.0 (5)
C13—C8—C9	120.9 (5)	O4—N2—C11	117.3 (5)
C13—C8—C7	123.2 (5)	O1—S1—O2	119.3 (3)
C9—C8—C7	115.9 (5)	O1—S1—N1	108.8 (3)
C8—C9—C10	120.1 (5)	O2—S1—N1	104.3 (2)
C8—C9—H9	120.0	O1—S1—C1	107.9 (3)
C10—C9—H9	120.0	O2—S1—C1	109.9 (2)
C11—C10—C9	117.8 (6)	N1—S1—C1	105.9 (3)
C11—C10—H10	121.1		
			
C6—C1—C2—C3	−0.3 (8)	C10—C11—C12—C13	−0.6 (10)
S1—C1—C2—C3	−175.1 (5)	N2—C11—C12—C13	−178.1 (5)
C6—C1—C2—C14	−178.4 (6)	C9—C8—C13—C12	−2.2 (9)
S1—C1—C2—C14	6.7 (8)	C7—C8—C13—C12	178.4 (6)
C1—C2—C3—C4	1.4 (10)	C11—C12—C13—C8	1.2 (9)
C14—C2—C3—C4	179.6 (6)	O3—C7—N1—S1	0.5 (8)
C2—C3—C4—C5	−2.8 (11)	C8—C7—N1—S1	−178.6 (4)
C3—C4—C5—C6	3.0 (11)	C12—C11—N2—O5	7.1 (8)
C2—C1—C6—C5	0.7 (9)	C10—C11—N2—O5	−170.5 (6)
S1—C1—C6—C5	175.7 (5)	C12—C11—N2—O4	−172.3 (6)
C4—C5—C6—C1	−1.9 (10)	C10—C11—N2—O4	10.1 (9)
O3—C7—C8—C13	160.5 (6)	C7—N1—S1—O1	−53.9 (5)
N1—C7—C8—C13	−20.4 (8)	C7—N1—S1—O2	177.8 (4)
O3—C7—C8—C9	−18.9 (9)	C7—N1—S1—C1	61.8 (5)
N1—C7—C8—C9	160.3 (5)	C6—C1—S1—O1	14.8 (5)
C13—C8—C9—C10	2.6 (9)	C2—C1—S1—O1	−170.0 (4)
C7—C8—C9—C10	−178.0 (5)	C6—C1—S1—O2	146.4 (5)
C8—C9—C10—C11	−2.0 (10)	C2—C1—S1—O2	−38.4 (6)
C9—C10—C11—C12	1.0 (10)	C6—C1—S1—N1	−101.5 (5)
C9—C10—C11—N2	178.5 (6)	C2—C1—S1—N1	73.6 (5)

### Hydrogen-bond geometry (Å, °)

**Table:**

*D*—H···*A*	*D*—H	H···*A*	*D*···*A*	*D*—H···*A*
N1—H1N···O2^i^	0.87 (3)	2.12 (4)	2.992 (6)	174 (5)

Symmetry codes: (i) −*x*+2, *y*+1/2, −*z*.
